# Long-term impact of a senior volunteer-driven frailty check programme on care dependency and expenditures: a 10-year population-based cohort study in Japan

**DOI:** 10.1093/ageing/afag161

**Published:** 2026-06-08

**Authors:** Tomoki Tanaka, Yasuyo Yoshizawa, Weida Lyu, Katsuya Iijima

**Affiliations:** Institute of Gerontology, The University of Tokyo, Bunkyo-ku, Tokyo, Japan; Institute for Future Initiatives, The University of Tokyo, Bunkyo-ku, Tokyo, Japan; Kashiwa City Hall, Kashiwa, Chiba, Japan; Institute of Gerontology, The University of Tokyo, Bunkyo-ku, Tokyo, Japan; Kashiwa City Hall, Kashiwa, Chiba, Japan; Department of Healthy Life Expectancy, Juntendo University, Bunkyo-ku, Tokyo, Japan; Institute of Gerontology, The University of Tokyo, Bunkyo-ku, Tokyo, Japan; Institute of Gerontology, The University of Tokyo, Bunkyo-ku, Tokyo, Japan; Institute for Future Initiatives, The University of Tokyo, Bunkyo-ku, Tokyo, Japan

**Keywords:** volunteer-driven, long-term care, frailty check programme, Japan, older people

## Abstract

**Background:**

Although volunteer-driven, community-based interventions to prevent long-term care (LTC) dependency have shown promise, long-term evidence of their effectiveness and economic impact remains scarce. We aimed to evaluate the association of a volunteer-driven frailty check (FC) programme with LTC certification and benefit expenditures.

**Methods:**

We conducted a 10-year retrospective, population-based cohort study in a municipality in Japan. Of 59 812 residents aged ≥65 years (2015–2024), FC participants were matched 1:1 with non-participants separately within each year using propensity scores, and the yearly matched sets were pooled for analysis, yielding 2672 participants and 2672 matched controls. Outcomes were incident LTC certification and annual per-capita LTC expenditures, analysed using Cox proportional hazards and generalised linear mixed-effects models.

**Results:**

Among 5344 matched individuals (mean age 73.5 ± 4.9 years), 16.8% of participants and 19.8% of controls received LTC certification [hazard ratio 0.85, 95% confidence interval (CI) 0.75–0.97; absolute risk reduction 3.0%; number needed to treat 33]. Annual per-capita LTC expenditures were consistently lower among participants, with the gap widening over time (cost ratio 0.66, 95% CI 0.51–0.84), yielding cumulative savings of ~JPY443 000 per participant over 10 years. Exploratory analyses indicated slower growth in combined medical and LTC expenditures.

**Conclusion:**

This programme was associated with lower LTC dependency and slower growth in combined medical and long-term care expenditures. Implemented in 106 municipalities in Japan, this volunteer-led, community-based model may offer a low-cost, scalable intervention to promote healthy ageing and sustainability in super-aged societies, with relevance for countries facing population ageing and fiscal constraints.

## Key points

- This study examined a volunteer-led frailty-check programme and its association with long-term care (LTC) certification and expenditures.
- This low-cost, community-based intervention was associated with delayed LTC dependency and reduced long-term care expenditures.
- Exploratory analyses showed slower growth in combined medical and LTC costs among participants.
- The programme has scaled to 106 municipalities in Japan, indicating feasibility for routine community prevention.
- This study provides real-world evidence supporting scalable, community-led models for healthy ageing

## Introduction

Global population ageing is reshaping healthcare, social care and welfare systems, posing significant challenges for policymakers and practitioners. In Japan—one of the world’s most rapidly ageing societies—the proportion of individuals aged 65 years or older is projected to exceed 35% by 2040, with those aged 75 years or older increasing even faster [1]. In Japan, rising care needs coincide with shrinking human resources. These trends are expected to culminate in the ‘2040 problem’, characterised by increasing care needs, workforce shortages, diminishing community resources, social isolation and regional population decline—collectively termed ‘community shrinkage’. This shift threatens the fiscal sustainability of long-term care (LTC) insurance (LTCI) and challenges healthcare system capacity, particularly amid shortages of healthcare professionals. Consequently, preventing or delaying LTC dependency has become a critical societal and clinical priority [2, 3].

Frailty is a key, modifiable risk factor in this context [4]. Defined as a multidimensional syndrome involving declines in physical, psychological and social functions, it increases disability risk, healthcare costs and mortality [5–8]. Frailty is potentially reversible when detected early, highlighting the importance of timely intervention. However, clinic-based screening and rehabilitation programmes, although beneficial individually, have limited scalability, cost-effectiveness and sustainability, particularly in resource-constrained settings [9, 10]. Accordingly, attention has shifted towards community-based, volunteer-driven strategies that empower older adults, promote mutual support and strengthen social cohesion [11, 12].

Aligned with Japan’s ‘community-based integrated care system’, a volunteer-driven frailty check (FC) programme was launched in 2015 in the study municipality. Developed collaboratively by municipal authorities, academic institutions and trained volunteers, the programme combines multidomain frailty screening, visual feedback and group discussion. Individuals identified as at risk are referred to municipal preventive services such as exercise classes, nutrition counselling and community activities [13]. This resident-led model is informed by social cognitive theory to promote behaviour change and collective efficacy while conserving medical resources in workforce-constrained societies [14].

Qualitative and small-scale studies suggest that such interventions improve health literacy, sense of purpose, social connection and sustainable behaviour change [13]. They may also restore social capital and shift older adults from passive recipients to active community contributors [14]. Recent evidence shows that FC results are associated with subsequent disability and mortality, supporting its prognostic validity [15]. However, prior studies often lacked sufficient follow-up, rigour or generalisability [16]. Consequently, robust large-scale evidence on long-term effects on LTC certification and expenditures remains limited.

This 10-year population-based cohort study examines the volunteer-driven FC programme in the study municipality and its association with LTC certification and expenditures. The findings highlight the potential of community-led models to reduce disability, contain costs, preserve healthcare resources and strengthen resilience in super-aged societies.

## Methods

### Study design and data sources

This retrospective, population-based cohort study leveraged comprehensive administrative data from the study municipality. Analysis included anonymised LTC insurance claims, certification records and healthcare utilisation data spanning May 2015 to December 2024. This study was approved by the ethics committee of The University of Tokyo (approval number: #23-161).

### Study population

The cohort included all residents of the study municipality aged 65 years or older as of May 2015 and registered in the city’s LTCI system. The FC programme was implemented across all 21 districts of the municipality. To establish a baseline of functional independence and avoid reverse causation, individuals with pre-existing LTC certification at baseline were excluded. Individuals who had already died, relocated outside the city, started receiving public assistance or lost their insurance eligibility before cohort entry were also excluded, as were those with incomplete or inconsistent administrative records. In contrast, individuals who died or lost their insurance eligibility after cohort entry were not excluded from the cohort; however, they were censored at the time of those events in the time-to-event analyses. After applying these criteria, the final analytic sample comprised 59 812 individuals (Supplementary Appendix Figure 1).

### Exposure

The volunteer-driven FC programme, initiated in 2015 as part of the study municipality’s integrated community care initiatives, is a resident-led community intervention designed for early frailty detection and health behaviour change (Supplementary Appendix Figure 2) [17–19]. The programme comprises multidomain frailty screening delivered by trained senior volunteers at community venues, with visual feedback and referral of high-risk individuals to municipal preventive services [17–21]. For this analysis, exposure was defined as participation in at least one FC session between May 2015 and December 2023. This definition reflects the programme’s real-world implementation, where initial attendance serves as the entry point to risk feedback, peer discussion and referral pathways. For the primary analysis, FC participation was operationalised within annual matched sets. In each year, individuals who newly participated in the FC programme were identified and matched with non-participants from the same source population using propensity scores estimated from pre-exposure characteristics. Subsequently, the yearly matched sets were pooled for analysis. Because the programme recommends follow-up approximately every 6 months, we additionally examined repeat participation patterns (eg, one-time vs repeat participation) to evaluate sustained engagement.

### Control group and matching

Propensity score matching minimised selection bias and confounding, producing a comparable control group of non-participants. Propensity scores were separately estimated within each year using a logistic regression model including age, sex, medical history (hypertension, diabetes, dyslipidaemia, cerebrovascular disease, ischaemic heart disease, chronic kidney disease, dementia and musculoskeletal system and connective tissue diseases), Charlson Comorbidity Index [22], and prior-year healthcare expenditures. Controls were matched to participants in a 1:1 ratio using a nearest-neighbour algorithm with a calliper width of 0.2 standard deviations of the logit of the propensity score. Covariate balance in the matched cohort was confirmed by standardised mean differences of less than 0.1 for all baseline variables. Then, matched participant–control sets created within each year were then pooled to form the final analytic cohort.

### Outcomes

The primary outcomes were (i) incidence of LTC certification, defined in Japan as the formal eligibility determination for LTCI benefits by municipal approval boards, based on standardised assessments of physical and cognitive function, and (ii) annual per-capita LTC benefit expenditures (in Japanese yen). For the analysis of incident LTC certification, follow-up in the pooled annual matched cohort continued until the earliest event among LTC certification, death, loss of insurance eligibility or the end of the observation period (December 2024). Thus, death and loss of insurance eligibility during follow-up were treated as censoring events. Because death precludes subsequent LTC certification, it was also considered a competing event at the time of result interpretation. LTCI services in Japan are provided to adults aged 40 years or older who are eligible for benefits in cases of physical or mental disability [23]. A public LTCI receipt database in the study municipality was employed in the current study [24]. Functional disability certification occurred when an individual, previously independent at the time of the health check-up survey, was newly certified as requiring LTC (care level 1 or more). The period of independence was defined as the interval between the date of the health check-up and date of certification [25].

### Statistical analysis

Statistical analyses were performed using IBM SPSS Statistics, version 29.0 (IBM Corp, Armonk, NY, USA). Statistical significance was set at *P* < .05. Data are presented as mean (±SD) or median [interquartile range (IQR)] for quantitative measures and as the number of participants (percentage) for qualitative measures.

The main analyses were conducted in the pooled cohort generated through annual 1:1 propensity score matching. Cox proportional hazards regression models stratified by matched pairs estimated hazard ratios (HRs) and 95% confidence intervals (CIs) for the time to LTC certification, adjusting for potential confounders. To examine temporal patterns, annual HRs were additionally summarised according to the year of annual matched analysis. Annual per-capita LTC expenditures were analysed using generalised linear mixed-effects models with a gamma distribution and log link to account for repeated yearly observations within individuals. These models included terms for year and FC participation status along with their interaction, and exponentiated coefficients are presented as cost ratios with 95% CIs. Parallel models were fitted for one-time versus repeat participation and, exploratorily, for combined medical and LTC expenditures. Repeated participation patterns were descriptively summarised. Absolute risk reduction (ARR), number needed to treat (NNT = 1/ARR), and cost differences were calculated to illustrate clinical significance. Sensitivity analyses included subgroup comparisons (by sex, age group and repeat participation).

To assess external validity, area-level analyses were also conducted across the study municipality’s 21 community districts to examine the consistency of programme effects on LTC certification and expenditures. Lifestyle factors (exercise and dietary habits) were explored only within participants.

## Results

Annual 1:1 propensity score matching yielded a pooled analytic cohort of 5344 individuals, including 2672 FC participants and 2672 matched controls (Supplementary Appendix  Figure 1). Baseline characteristics were well balanced between groups, with all standardised mean differences remaining below 0.1 in the pooled matched cohort (Table 1; Supplementary Appendix Tables 1 and 2). The mean age was similar between groups (73.5 vs. 73.5 years), and women comprised ~75% participants in each group (74.4% vs. 75.0%). The prevalence of major comorbidities and prior healthcare expenditures was also comparable between groups. Across all participants (*n* = 2672), 64.9% attended once, 18.5% attended twice, 8.6% three times, 3.6% four times and 4.4% attended five or more times. Among repeat participants (*n* = 939), the median number of sessions was 2.0 (IQR 2.0–3.0; range 2–11), and the median time to second participation was 181 days (IQR 154–243).

**Table 1:** Baseline characteristics of the matched cohort.

Characteristic	Non-participants	FC participants	*P*
Number of participants	2672	2672	
**Basic attribute**			
Age, years	73.5 (± 4.9)	73.5 (± 4.9)	.948
65–74 years	1654 (61.9%)	1647 (61.6%)	
≥75 years	1018 (38.1%)	1025 (38.4%)	
Sex, female	1989 (74.4%)	2004 (75.0%)	.865
**Medical history** ^**a**^			
Hypertension	1899 (71.0%)	1890 (70.7%)	.895
Diabetes	1086 (40.6%)	1085 (40.6%)	1.000
Dyslipidaemia	890 (33.3%)	885 (33.1%)	.865
Cerebrovascular disease	861 (32.2%)	874 (32.7%)	.974
Ischaemic heart disease	650 (24.3%)	674 (25.2%)	.653
Chronic kidney disease	221 (8.3%)	218 (8.1%)	.761
Dementia	40 (1.5%)	39 (1.5%)	.987
Musculoskeletal system and connective tissue diseases	1793 (67.1%)	1758 (65.8%)	.488
CCI score, median (IQR)	1.0 (0.0–3.0)	1.0 (0.0–3.0)	.991
**Medical costs (JPY)**			
Total costs^b^	470.2 (± 402.9)	463.1 (± 424.1)	.456
FY2015	371.1 (± 561.3)	363.8 (± 577.8)	.777
FY2016	392.7 (± 664.3)	400.8 (± 553.6)	.766
FY2017	391.6 (± 617.4)	398.3 (± 608.5)	.811
FY2018	448.3 (± 738.4)	459.2 (± 676.8)	.436
FY2019	397.2 (± 793.2)	398.3 (± 779.8)	.890
FY2020	451.8 (± 954.2)	451.6 (± 930.7)	.882
FY2021	499.3 (± 991.7)	492.8 (± 932.4)	.675
FY2022	504.2 (± 982.6)	541.7 (± 960.8)	.226
FY2023	531.2 (± 1032.2)	592.3 (± 1199.0)	.346

CCI, Charlson Comorbidity Index; FY, fiscal year.

^a^Based on outpatient/inpatient records FY2015–FY2023.

^b^Average per year (1000 yen), FY2015–FY2023.

### LTC certification rate

**Figure 1:**
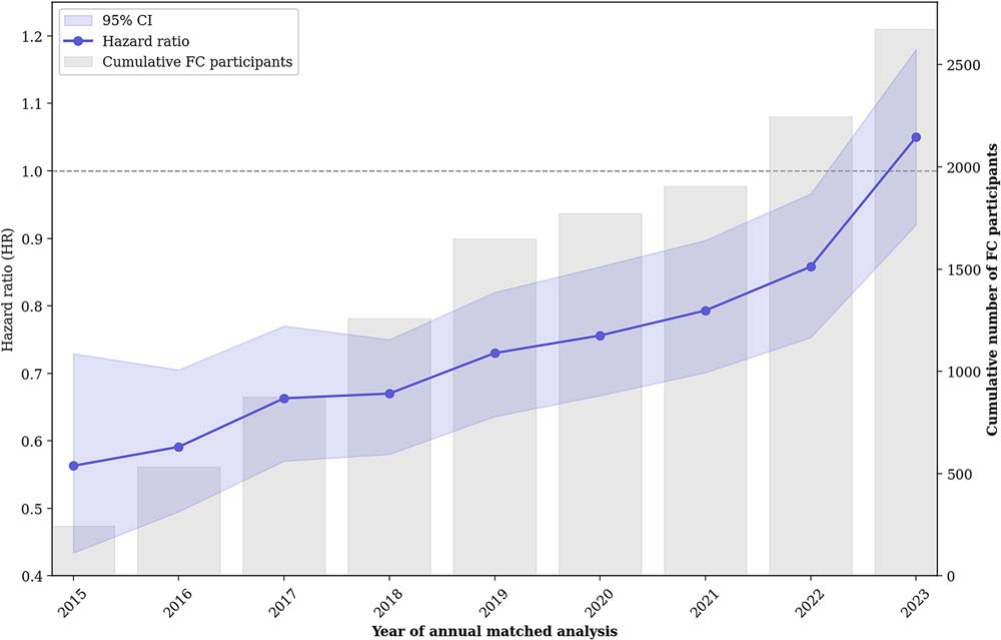
Annual HRs for incident long-term care certification and cumulative number of FC participants. Notes: The figure shows the HRs and 95% CIs for incident long-term care certification according to the year of annual matched analysis, together with the cumulative number of FC participants. FC participants were matched 1:1 with non-participants within each year using propensity scores, and the yearly matched sets were pooled for analysis.

Over 10 years, LTC certification occurred in 19.8% controls (530/2672) and 16.8% participants (450/2672); these findings yielded an ARR of 3.0% (95% CI: 0.9 to 5.1%) and NNT of 33 (95% CI: 20 to 109). Participation was associated with a lower hazard of LTC certification (HR 0.85, 95% CI 0.75–0.97). Annual HRs showed that this association was the strongest in earlier years and attenuated over time (Figure 1): HR was lowest in 2015 (0.56, 95% CI 0.43–0.73) and no longer showed a statistically significant association in 2023 (1.05, 95% CI 0.92–1.18).

Dose–response analyses showed lower hazards for one-time participants (adjusted HR 0.91, 95% CI 0.83–0.98) and repeat participants (adjusted HR 0.69, 95% CI 0.57–0.84; Table 2).

**Table 2:** Association of participation in the FC-up programme with HRs for new LTC certification estimated by Cox proportional hazards models.

Comparison	Exposure	*n/N* (%)	Adjusted HR (95% CI)	*P*
Participants vs. non-participants	Non-participants	530/2672 (19.8%)	1.00 (reference)	
	Participants (time-dependent)	450/2672 (16.8%)	0.85 (0.75 to 0.97)	.023
Dose–response	Non-participants	530/2672 (19.8%)	1.00 (reference)	
	Just once (time-dependent)	302/1733 (17.4%)	0.91 (0.83 to 0.98)	.043
	Repeaters (time-dependent)	148/939 (15.8%)	0.69 (0.57 to 0.84)	<.001

Notes: Analyses were conducted in a propensity score–matched cohort (1:1 matching) matched on baseline age, sex, comorbidities and total healthcare costs. HRs were estimated using a Cox proportional hazards model.

### LTC expenditures

**Figure 2:**
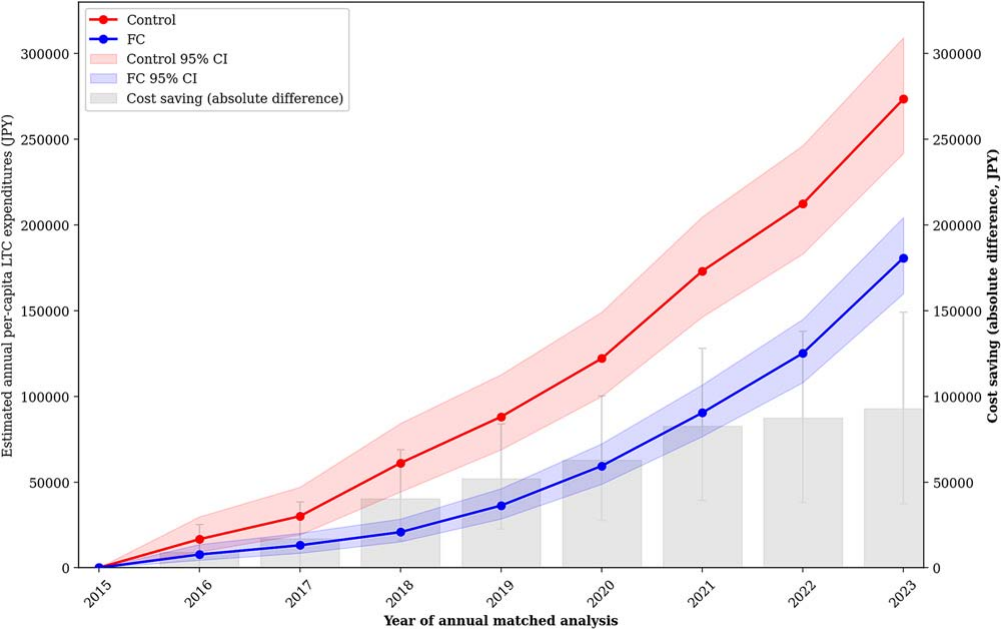
Estimated annual per-capita long-term care expenditures by participation in the FC programme. Notes: The figure presents the estimated annual per-capita long-term care expenditures and 95% CIs for FC participants and matched controls across duration (years) of annual matched analysis. Bars represent the absolute difference in estimated expenditures between the two groups.

Annual per-capita LTC expenditures were consistently lower among participants than non-participants, with a widening gap over time (Figure 2). One-time and repeat participants also showed lower expenditures than did controls, with stronger associations among repeat participants (Table 3). Dose–response patterns were evident: one-time participants incurred lower costs than did non-participants (cost ratio 0.58, 95% CI 0.50–0.68), and repeat participants showed the greatest reduction (cost ratio 0.35, 95% CI 0.30–0.41). Interaction terms also supported slower cost growth over time for both one-time participants (time × one-time participation: 0.88, 95% CI 0.82–0.93) and repeat participants (time × repeat participation: 0.87, 95% CI 0.83–0.94).

**Table 3:** Association of participation in the FC-up programme with annual per-capita LTC expenditures.

Comparison	Effect	Estimated annual per-capita LTC expenditures, mean (SE)	β (SE)	Cost ratio (95% CI)	*P*
Participants vs non-participants	Time	–	0.43 (0.02)	1.53 (1.47 to 1.59)	<.001
	Non-participants	84 568 (5163)	0.00	1.00 (reference)	
	Participants (time-varying)	43 338 (1940)	−0.52 (0.13)	0.66 (0.51 to 0.84)	<.001
	Time* Participants (time-varying)		−0.17 (0.04)	0.84 (0.78 to 0.91)	<.001
Dose–response	Time	–	0.43 (0.02)	1.53 (1.48 to 1.59)	<.001
	Non-participants	84 220 (5173)	0.00	1.00 (reference)	
	Just once (time-varying)	51 539 (2821)	−0.55 (0.08)	0.58 (0.50 to 0.68)	<.001
	Repeaters (time-varying)	21 235 (2015)	−1.19 (0.10)	0.35 (0.30 to 0.41)	<.001
	Time*Just once (time-varying)	–	−0.12 (0.04)	0.88 (0.82 to 0.93)	.002
	Time*Repeaters (time-varying)	–	−0.12 (0.03)	0.87 (0.83 to 0.94)	<.001

Notes: Analyses were conducted in a propensity score–matched cohort (1:1 matching) calculated by baseline age, sex, comorbidities and total healthcare costs. Ratios were estimated using generalised linear mixed-effects models with participation as a time-varying variable.

SE, standard error.

Exploratory analyses revealed a slower increase in total healthcare expenditures (medical + LTC) among participants than among non-participants (Supplementary  Appendix Table 3, Supplementary Appendix Figure 3). In exploratory models, participation was associated with lower total expenditures overall (cost ratio 0.84, 95% CI 0.75–0.94), whereas the time-by-participation interaction was not statistically significant overall (cost ratio 1.00, 95% CI 0.99–1.00).

### Sensitivity and subgroup analyses

Subgroup analyses suggested somewhat stronger associations among women and those aged 65–74 years (Supplementary Appendix Table 4). In addition, exploratory analyses suggested more favourable associations among participants with regular exercise and healthier dietary habits, although lifestyle data were unavailable for controls. Area-level analyses across the municipality’s 21 districts showed broadly consistent findings.

## Discussion

This study suggests that participation in a volunteer-driven FC programme was associated with a lower risk of LTC dependency and lower LTC-related expenditures over a 10-year period. Although the ARR was modest (ARR 3.0%; NNT 33), even small individual-level differences were associated with large cumulative savings at the population level. In super-aged societies facing increasing care demands and a declining healthcare workforce, resident-led models may serve as not only community-based preventive approaches but also mechanisms for conserving scarce professional resources.

Annual matched analyses identified the strongest preventive effect in the initial years after participation, with attenuation over time. It may reflect several non-mutually exclusive factors, including shorter follow-up among later entrants, ageing of the pooled cohort, changes in the composition of exposed and unexposed groups over time, maturation of the programme itself and broader secular changes in health and care systems. Notably, repeat participants consistently demonstrated lower HRs and greater cost savings than did non-participants and one-time participants; this suggested that repeated participation may be important for sustaining programme related benefits. Indeed, among repeat participants (*n* = 939), the median number of attendances was 2 (IQR 2–3; range 2–11), and the median interval to a second attendance was 181 days (IQR 154–243), consistent with the programme’s recommendation of an approximately 6-month follow-up period.

Plausible mechanisms for these outcomes include enhanced health awareness and motivation fostered through personalised risk feedback, which may prompt proactive, preventive actions. Group-based discussions and peer learning may cultivate social connectedness, collective efficacy and a sense of belonging, all of which are associated with improved functional outcomes, reduced frailty risk and diminished feelings of isolation [26–28]. Timely linkage of high-risk individuals to preventive services, such as exercise, nutrition and oral health programmes, may also facilitate early intervention before irreversible decline [29, 30]. Moreover, the programme may encourage a psychological shift from passive recipients to active community contributors, strengthening purpose, self-efficacy and social capital. These pathways align with emerging evidence that multidimensional frailty screening combined with social participation can improve health trajectories and enhance community resilience [28]. Recent findings have also demonstrated that FC results predict subsequent disability and mortality [15]; the present study extends this evidence by demonstrating favourable associations with lower LTC certification rates and expenditures among participants.

The apparent discrepancy between modest individual-level effects and substantial per-capita cost savings can be explained by the structure of Japan’s LTCI system, where even a small delay in certification leads to large financial differences due to high annual expenditures following certification. Thus, the observed ARR of 3.0% (NNT 33) translated into cumulative savings of ~¥443 000 per participant over the study period. Exploratory analyses suggested slower growth in combined healthcare expenditures among FC participants, despite similar baseline medical costs. This may reflect broader impacts, such as increased health awareness and timely referral to appropriate professional care, which can prevent costly medical deterioration. This interpretation is consistent with prior cohort evidence showing transitions into worse frailty states are associated with increased healthcare costs [8]. While these findings should be interpreted cautiously, they suggest that the programme’s fiscal benefits may extend beyond LTC savings. Mechanistically, the FC programme may operate through incremental gains in health awareness, behaviour and social participation, which may help maintain functional capacity and delay dependency onset.

From a policy perspective, the FC programme exemplifies a sustainable, scalable and culturally adaptable strategy that complements formal care systems and aligns with the principles of healthy ageing and social inclusion promoted by the World Health Organization’s ‘Decade of Healthy Ageing’ [31]. Reliance on trained resident volunteers reduces dependence on clinical professionals, particularly in countries facing shortages of physicians and nurses. The programme has expanded beyond the study municipality, with participation exceeding 50 000 older adults across 106 municipalities in Japan, demonstrating feasibility, acceptability and potential for policy uptake. However, benefits may diminish over time without continued engagement, emphasising the need for programme designs that encourage ongoing participation [32, 33]. Comparable programmes in other settings have shown promise, suggesting that this model can serve as a blueprint for resilient, inclusive communities globally [34]. Nevertheless, transferability may depend on local sociocultural norms, volunteer capacity and the availability of community-based preventive services and referral pathways.

Study strengths include a large population-based sample, long follow-up and use of administrative data capturing real-world LTC outcomes and expenditures, enhancing validity and policy relevance. An additional strength is that participants were matched to non-participants separately within each year using propensity scores, and the yearly matched sets were pooled for analysis, thereby improving alignment between programme participation and control selection. Limitations include residual confounding and selection bias, as participants may have been more health-conscious at baseline. Although propensity score matching balanced measured factors, unmeasured differences (e.g. motivation, social engagement or help-seeking behaviour) may remain. Accordingly, the observed associations should not be interpreted as definitively causal. In addition, although deaths and loss of insurance eligibility during follow-up were treated as censoring events, death also acts as a competing event for LTC certification and should be considered when interpreting the findings. As a resident-led initiative, variation in programme delivery may occur, highlighting the need for standardisation as scale grows. The absence of lifestyle data for controls limits interpretation of exploratory analyses, although within-participant subgroups suggest effects were not solely due to pre-existing differences. Area-level analyses across the study municipality’s 21 districts showed broadly consistent results, supporting external validity in urban–suburban Japan. Generalisability beyond similar sociocultural and healthcare settings remains limited. Future research should examine implementation fidelity, adaptation and long-term behavioural and psychosocial outcomes.

## Conclusions

Volunteer-driven frailty prevention through the FC programme is associated with lower LTC certification rates and reduced benefit expenditures over one decade. Beyond individual-level effects, the programme’s design demonstrates how modest yet scalable interventions may yield significant population-level savings. Exploratory analyses suggest a potential for attenuated growth in total healthcare expenditures, highlighting a broader fiscal relevance. Additionally, the programme’s community-empowering design can strengthen social capital, revitalise communities and foster inclusion in super-aged, shrinking societies. However, the observed associations were the strongest in earlier years and attenuated over time. These findings support programme designs that promote continued engagement, alongside mechanisms for standardised delivery and linkage to preventive services. With global population ageing, scalable, culturally adaptable interventions, such as the FC programme, may help older adults to age with dignity and autonomy, reduce societal costs and strengthen community resilience. Empowering communities to co-produce preventive care may offer a viable and transferable model for countries across various income levels that face rapid population ageing and those with constrained healthcare workforces.

## Supplementary Material

aa-25-3506-File004_afag161

## Data Availability

The data that support the findings of this study are not publicly available due to legal and ethical restrictions, as they include administrative long-term care and healthcare records managed by the municipality. Access to the data may be considered upon reasonable request, subject to approval by the data provider and the relevant ethics committees.
