# Supplementary material for: Long-term impact of a senior volunteer-driven frailty check programme on care dependency and expenditures: a 10-year population-based cohort study in Japan
Source: Age Ageing. 2026 Jun 8;55(6):afag161. doi: 10.1093/ageing/afag161 (PMC13245724; doi:10.1093/ageing/afag161)
Supplement: aa-25-3506-File004_afag161 [file aa-25-3506-file004_afag161.docx]

**APPENDIX**

**
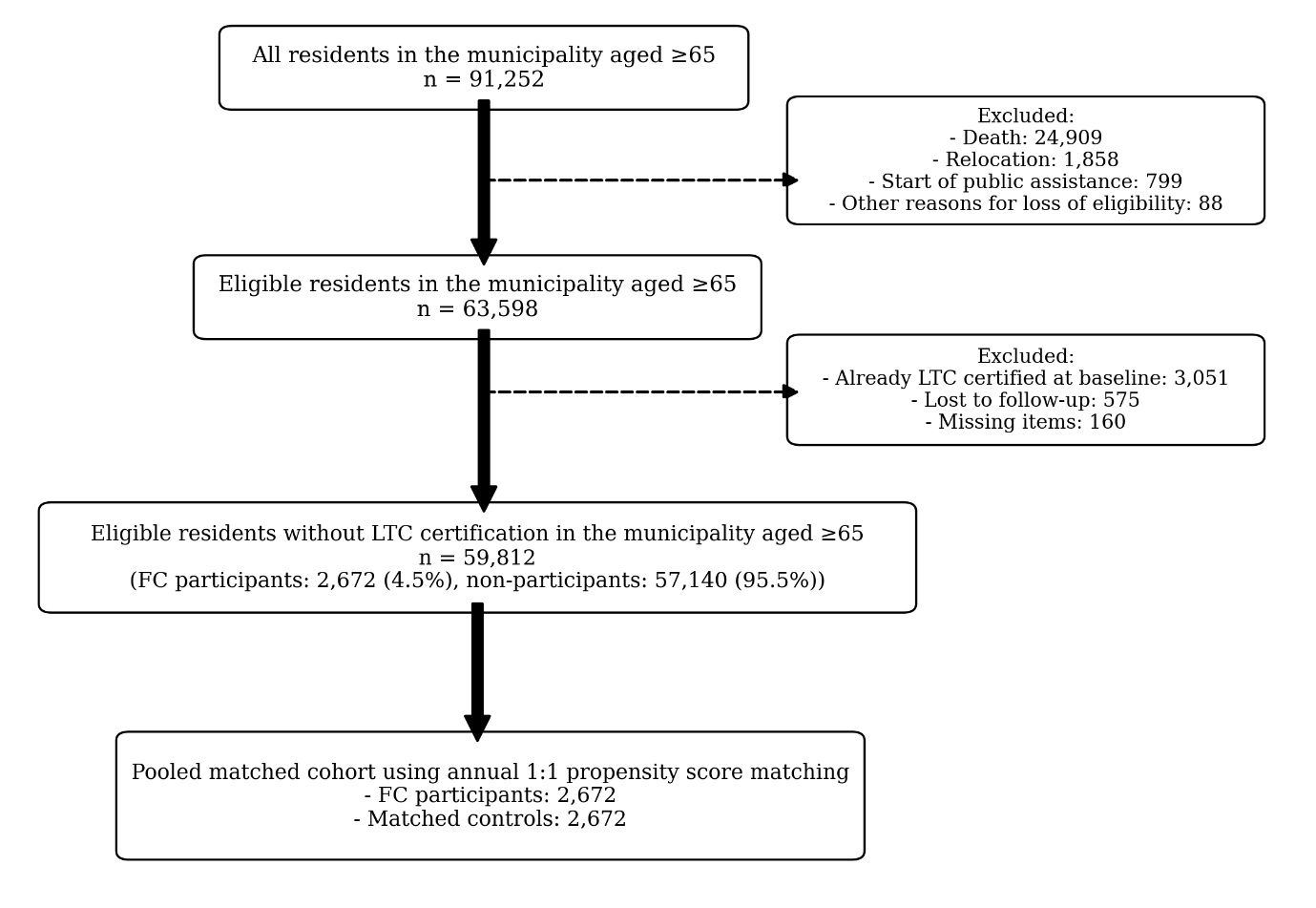
**

**Appendix Figure 1.** Study flow diagram for the pooled cohort created through annual propensity score matching.

*Notes:* The figure illustrates the selection of eligible residents aged 65 years or older in the study municipality, the exclusion criteria, and the derivation of the final pooled matched cohort. Frailty check participants were matched 1:1 with non-participants separately within each year using propensity scores, and the yearly matched sets were pooled for analysis.


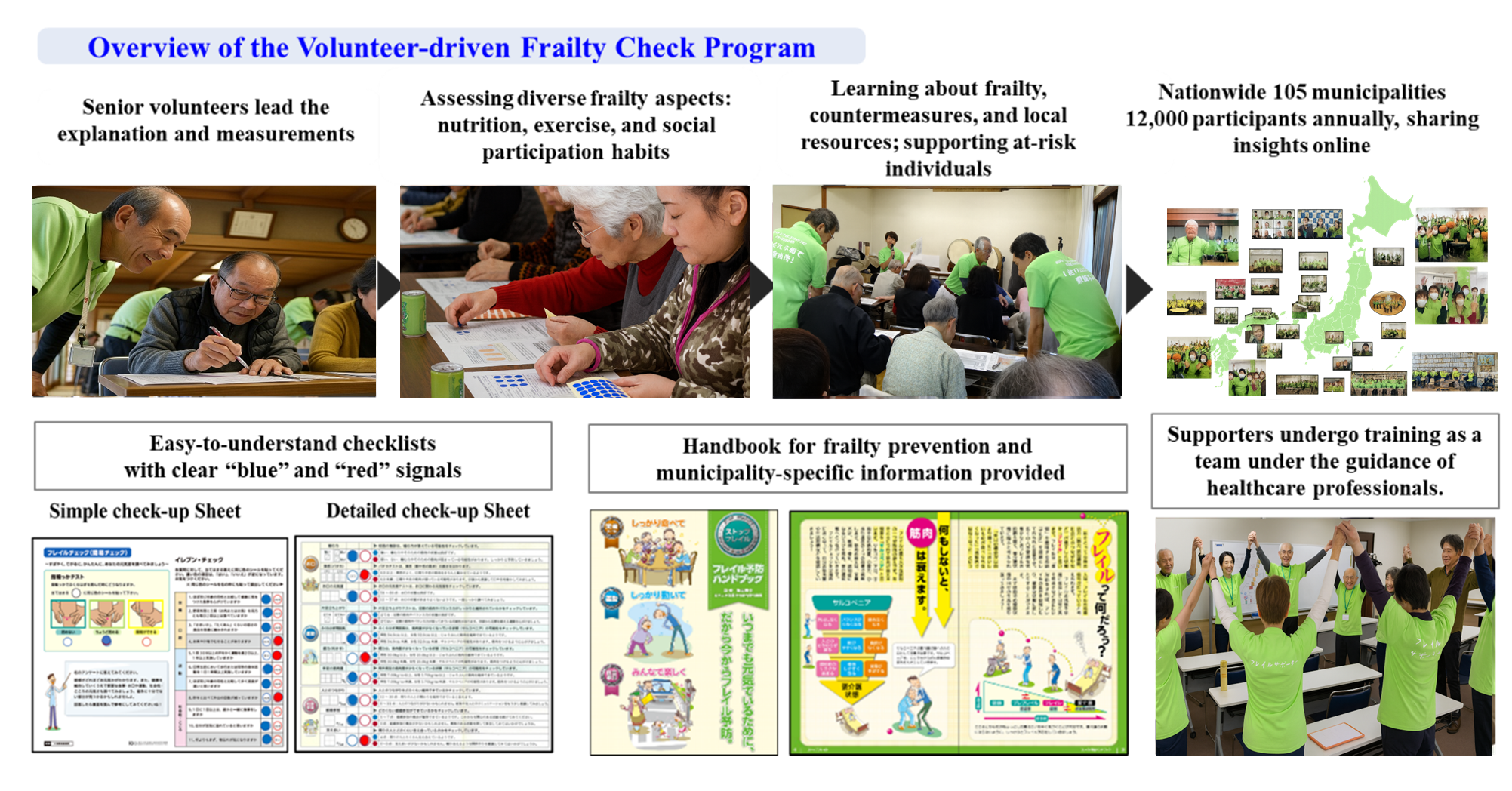


**Appendix Figure 2.** Volunteer-driven Frailty Check Program promoting awareness, engagement, and nationwide use

*Notes:* Frailty Check programmes aim to raise awareness of frailty by addressing nutrition, oral health, physical activity, and social factors. The programme uses a 22-item structured checklist combining self-reported items and equipment-based measurements. Participants visually mark results as “good” or “needs attention” using coloured stickers and receive personalised feedback and advice from trained senior supporters after the session. Group-based discussions facilitate shared learning and reflection. As of 2025, the programme is implemented in 106 municipalities across Japan at accessible community venues, and participation is voluntary following public announcement through local communication channels.

This illustrative image represents a community-based Frailty Check session. The image was generated using artificial intelligence based on anonymised reference photographs for illustrative purposes only. It does not depict real individuals, and no identifiable personal information is included.

Community salons allow participants to compare their results with standard reference values and to receive tailored guidance on preventive actions. Follow-up sessions held approximately every six months aim to reinforce awareness and support maintenance of independence. All volunteers are trained under professional supervision using standardised manuals, and calibration procedures conducted by healthcare professionals are applied to minimise measurement error. High-risk participants are referred to appropriate municipal preventive services, including exercise programmes, nutrition counselling, and social activity initiatives.


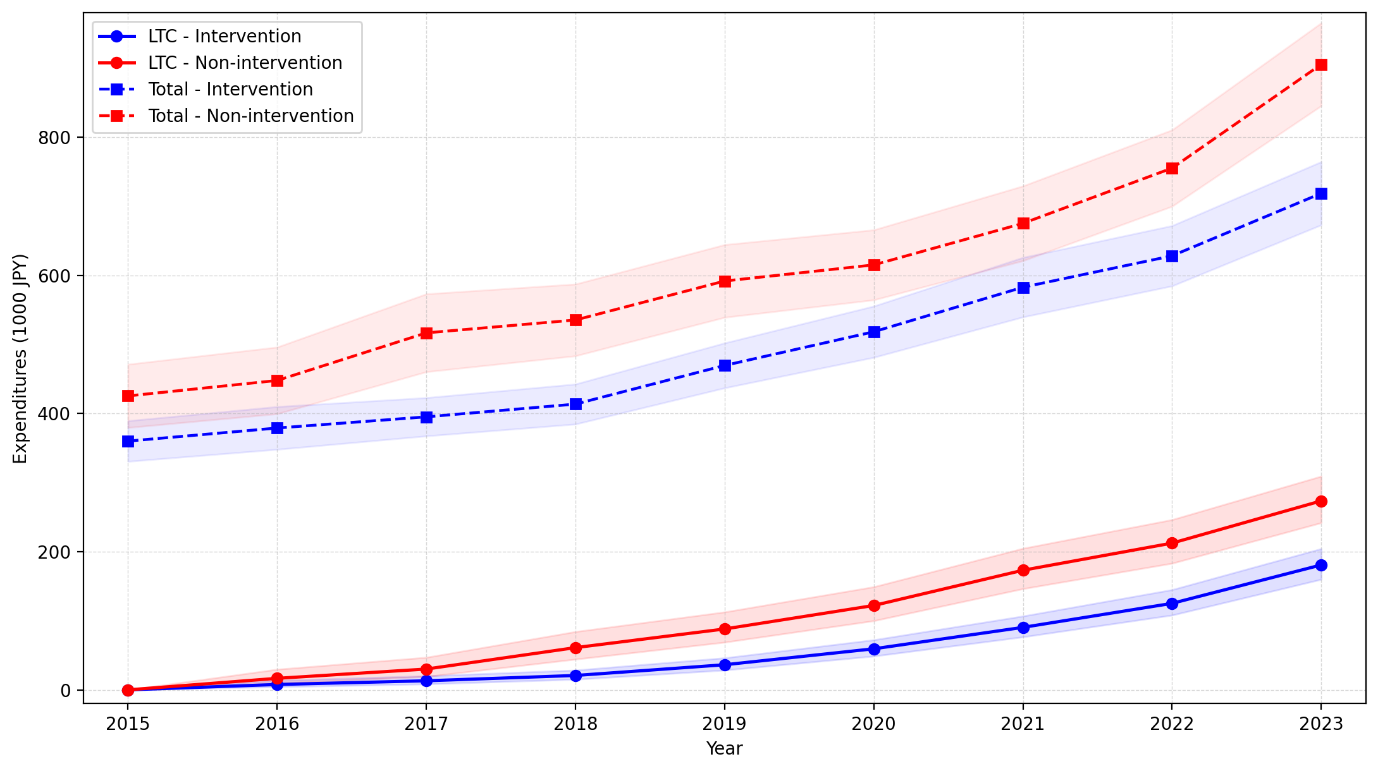


**Appendix Figure 3.** Appendix Figure 3. Estimated annual per-capita total and long-term care expenditures in frailty check participants and matched controls.

**Notes:** Lines indicate estimated annual per-capita expenditures for frailty check participants and matched controls, with shaded areas representing the 95% confidence intervals. Solid lines represent long-term care expenditures and dashed lines represent total expenditures. Expenditures are expressed in 1000 JPY. Analyses were conducted in a pooled cohort created through annual 1:1 propensity score matching.

**Appendix Table 1.** Baseline characteristics and standardised mean differences of annual matched cohorts (2015–2019) and the pooled matched cohort

|  | **2015** | | | **2016** | | | **2017** | | | **2018** | | | **2019** | | |
| --- | --- | --- | --- | --- | --- | --- | --- | --- | --- | --- | --- | --- | --- | --- | --- |
|  | **Control** | **FC participants** | **SMD** | **Control** | **FC participants** | **SMD** | **Control** | **FC participants** | **SMD** | **Control** | **FC participants** | **SMD** | **Control** | **FC participants** | **SMD** |
| No. of participants | 283 | 283 |  | 322 | 322 |  | 323 | 323 |  | 403 | 403 |  | 323 | 323 |  |
| Age | 74.5 (5.0) | 74.4 (5.5) | 0.020 | 74.4 (4.8) | 74.1 (5.1) | 0.000 | 74.5 (4.8) | 74.2 (5.0) | 0.021 | 74.6 (4.9) | 74.2 (5.4) | 0.021 | 73.6 (4.8) | 73.4 (4.6) | 0.021 |
| Sex, % of women | 75.1% | 74.6% | 0.007 | 79.1% | 78.6% | 0.005 | 74.5% | 74.9% | 0.009 | 78.8% | 79.9% | 0.009 | 74.3% | 74.9% | 0.007 |
| **Medical history** |  |  |  |  |  |  |  |  |  |  |  |  |  |  |  |
| Hypertension | 70.3% | 71.1% | 0.018 | 70.4% | 70.1% | 0.007 | 69.2% | 70.2% | 0.022 | 71.1% | 72.1% | 0.022 | 73.1% | 71.9% | 0.027 |
| Diabetes | 40.2% | 40.3% | 0.002 | 39.8% | 40.1% | 0.006 | 40.4% | 39.9% | 0.010 | 40.1% | 40.5% | 0.008 | 40.1% | 40.3% | 0.004 |
| Dyslipidaemia | 32.9% | 33.1% | 0.004 | 33.9% | 34.2% | 0.006 | 31.9% | 32.1% | 0.004 | 34.7% | 34.1% | 0.013 | 31.9% | 32.9% | 0.021 |
| Cerebrovascular disease | 31.9% | 32.1% | 0.004 | 32.2% | 32.6% | 0.009 | 32.2% | 32.9% | 0.015 | 32.1% | 33.9% | 0.038 | 32.2% | 32.7% | 0.011 |
| Ischaemic heart disease | 24.2% | 24.9% | 0.016 | 24.2% | 23.9% | 0.007 | 24.2% | 25.3% | 0.027 | 24.2% | 25.2% | 0.023 | 24.3% | 25.2% | 0.021 |
| Chronic kidney disease | 8.2% | 8.1% | 0.004 | 8.3% | 8.2% | 0.004 | 8.3% | 8.0% | 0.011 | 8.3% | 8.1% | 0.007 | 8.3% | 8.1% | 0.007 |
| Dementia | 1.5% | 1.5% | 0.000 | 1.5% | 1.5% | 0.000 | 1.9% | 1.4% | 0.040 | 1.7% | 1.6% | 0.008 | 1.8% | 1.4% | 0.032 |
| Musculoskeletal system and connective tissue diseases | 67.2% | 65.9% | 0.028 | 67.2% | 66.9% | 0.006 | 67.2% | 64.7% | 0.053 | 67.2% | 65.5% | 0.036 | 67.1% | 65.8% | 0.028 |
| CCI score, median (IQR) | 1.0 (0-3) | 1.0 (0-3) | - | 1.0 (0-3) | 1.0 (0-3) | - | 1.0 (0-3) | 1.0 (0-3) | - | 1.0 (0-3) | 1.0 (0-3) | - | 1.0 (0-3) | 1.0 (0-3) | - |

**Abbreviations:** FC, frailty check-up; SEM, standard error of the mean; CI, confidence interval; JPY, Japanese yen.

**Appendix Table 2.** Baseline characteristics and standardised mean differences of annual matched cohorts (2020–2023) and the pooled matched cohort

|  | **2020** | | | **2021** | | | **2022** | | | **2023** | | | **Total** | | |
| --- | --- | --- | --- | --- | --- | --- | --- | --- | --- | --- | --- | --- | --- | --- | --- |
|  | **Control** | **FC participants** | **SMD** | **Control** | **FC participants** | **SMD** | **Control** | **FC participants** | **SMD** | **Control** | **FC participants** | **SMD** | **Control** | **FC participants** | **SMD** |
| No. of participants | 125 | 125 |  | 170 | 170 |  | 349 | 349 |  | 374 | 374 |  | 2,672 | 2,672 |  |
| Age | 73.1 (4.9) | 72.9 (4.6) | 0.000 | 72.5 (4.6) | 72.2 (3.9) | 0.000 | 73.1 (4.9) | 72.8 (4.7) | 0.020 | 72.7 (4.9) | 72.2 (4.0) | 0.021 | 73.5 (4.9) | 73.5 (4.9) | 0.000 |
| Sex, % of women | 74.5% | 75.2% | 0.002 | 68.5% | 67.1% | 0.002 | 75.3% | 76.8% | 0.002 | 70.2% | 69.5% | 0.021 | 74.4% | 75.0% | 0.014 |
| **Medical history** |  |  |  |  |  |  |  |  |  |  |  |  |  |  |  |
| Hypertension | 71.1% | 70.6% | 0.011 | 70.1% | 69.5% | 0.013 | 73.0% | 72.1% | 0.020 | 71.2% | 71.0% | 0.004 | 71.0% | 70.7% | 0.007 |
| Diabetes | 39.7% | 40.1% | 0.008 | 40.2% | 40.4% | 0.004 | 39.1% | 40.2% | 0.022 | 40.2% | 40.3% | 0.002 | 40.6% | 40.6% | 0.000 |
| Dyslipidaemia | 33.2% | 33.3% | 0.002 | 33.2% | 36.1% | 0.061 | 31.8% | 32.1% | 0.006 | 33.8% | 34.2% | 0.008 | 33.3% | 33.1% | 0.004 |
| Cerebrovascular disease | 32.1% | 32.5% | 0.009 | 32.6% | 32.4% | 0.004 | 33.1% | 33.2% | 0.002 | 29.6% | 29.9% | 0.007 | 32.2% | 32.7% | 0.011 |
| Ischaemic heart disease | 25.1% | 26.1% | 0.023 | 24.2% | 25.6% | 0.032 | 23.9% | 23.8% | 0.002 | 24.1% | 24.9% | 0.019 | 24.3% | 25.2% | 0.021 |
| Chronic kidney disease | 7.9% | 7.4% | 0.019 | 8.1% | 8.1% | 0.000 | 7.8% | 7.6% | 0.008 | 8.4% | 8.2% | 0.007 | 8.3% | 8.1% | 0.007 |
| Dementia | 1.4% | 1.5% | 0.008 | 1.6% | 1.4% | 0.016 | 1.5% | 1.4% | 0.008 | 1.6% | 1.4% | 0.016 | 1.5% | 1.5% | 0.007 |
| Musculoskeletal system and connective tissue diseases | 66.5% | 66.1% | 0.008 | 67.2% | 67.1% | 0.002 | 66.9% | 66.1% | 0.017 | 66.9% | 68.2% | 0.028 | 67.1% | 65.8% | 0.000 |
| CCI score, median (IQR) | 1.0 (0-3) | 1.0 (0-3) | - | 1.0 (0-3) | 1.0 (0-3) | - | 1.0 (0-3) | 1.0 (0-3) | - | 1.0 (0-3) | 1.0 (0-3) | - | 1.0 (0-3) | 1.0 (0-3) | 0.028 |

**Abbreviations:** FC, frailty check-up; SEM, standard error of the mean; CI, confidence interval; JPY, Japanese yen.

**Appendix Table 3.** Association of participation in the Frailty Check programme with annual per-capita total healthcare expenditures

| Comparison | Estimated annual per-capita total healthcare expenditures, mean (SEM), 1000 JPY | β (SEM) | Cost ratio (95% CI) | *P* value |
| --- | --- | --- | --- | --- |
| **Participants or non-participants** |  |  |  |  |
| Time | – | 0.12 (0.01) | 1.13 (1.11 to 1.14) | < 0.001 |
| Non-participants | 522.04 (6.08) | 0.00 | 1.00 (reference) | – |
| Participants (time-varying) | 621.53 (9.74) | –0.17 (0.06) | 0.84 (0.75 to 0.94) | 0.003 |
| Time × Participants (time-varying) | – | 0.004 (0.009) | 1.00 (0.99 to 1.00) | 0.704 |
| **Dose–response** |  |  |  |  |
| Time | – | 0.12 (0.01) | 1.13 (1.12 to 1.15) | < 0.001 |
| Non-participants | 628.36 (9.42) | 0.00 | 1.00 (reference) | – |
| One-time participants (time-varying) | 535.70 (9.70) | –0.13 (0.08) | 0.88 (0.75 to 1.02) | 0.088 |
| Repeat participants (time-varying) | 481.05 (11.5) | -0.19 (0.07) | 0.83 (0.73 to 0.94) | 0.004 |
| Time × One-time (time-varying) | – | 0.01 (0.01) | 1.01 (0.98 to 1.03) | 0.639 |
| Time × Repeaters (time-varying) | – | –0.02 (0.01) | 0.96 (0.94 to 0.99) | 0.043 |

Analyses were conducted in a propensity score–matched cohort (1:1 matching) calculated by baseline age, sex, comorbidities, and total healthcare costs. Ratios were estimated using generalised linear mixed-effects models with participation as a time-varying variable.

**Abbreviations:** SEM, standard error of the mean; CI, confidence interval; JPY, Japanese yen.

**Appendix Table 4.** Sensitivity analysis: Adjusted hazard ratios for new LTC certification estimated by time-dependent Cox proportional hazards models in participants versus non-participants by subgroup

| **Subgroup** | **Exposure** | ***n/N*** | **Adjusted HR (95% CI)** | ***P* value** |
| --- | --- | --- | --- | --- |
| Male | Non-participants | 125/683 | 1.00 (reference) |  |
|  | Participants (time-dependent) | 114/668 | 0.93 (0.85 to 0.99) | 0.049 |
| Female | Non-participants | 405/1,989 | 1.00 (reference) |  |
|  | Participants (time-dependent) | 336/2,004 | 0.85 (0.73 to 0.96) | 0.003 |
| Young-old (65–74) | Non-participants | 187/1,654 | 1.00 (reference) |  |
|  | Participants (time-dependent) | 149/1,647 | 0.84 (0.76 to 0.92) | 0.015 |
| Old-old (≥75) | Non-participants | 343/1,018 | 1.00 (reference) |  |
|  | Participants (time-dependent) | 251/1,025 | 0.71 (0.65 to 0.78) | <0.001 |
| Exercise habit* | Non-participants | 530/2,672 | 1.00 (reference) |  |
|  | Participants (time-dependent) without exercise habit | 145/713 | 1.03 (0.96 to 1.12) | 0.312 |
|  | Participants (time-dependent) with exercise habit | 305/1,959 | 0.80 (0.73 to 0.87) | < 0.001 |
| Healthier dietary habits** | Non-participants | 530/2,672 | 1.00 (reference) |  |
|  | Participants (time-dependent) with unhealthier dietary habit | 89/438 | 1.04 (0.95 to 1.12) | 0.289 |
|  | Participants (time-dependent) with healthier dietary habit | 361/2,234 | 0.82 (0.74 to 0.90) | 0.003 |

**Abbreviations:** HR, hazard ratio; LTC, long-term care; CI, confidence interval.

*Exercise habit was assessed using the self-administered question: ‘*Is there any exercise that you take for at least 30 min a day, twice a week or more, that you have engaged in for at least one year’?* (Yes/No).

**Healthier dietary habits were assessed using the self-administered question: ‘*Do you eat vegetables and either meat or fish daily’?* (Yes/No).
